# Comprehensive Analysis of the Expression Profiles of Hepatic lncRNAs in the Mouse Model of Alcoholic Liver Disease

**DOI:** 10.3389/fphar.2021.709287

**Published:** 2021-07-29

**Authors:** Xiaobing Dou, Wenwen Yang, Qinchao Ding, Qiang Han, Qianyu Qian, Zhongyan Du, Yibin Fan, Cui Wang, Songtao Li

**Affiliations:** ^1^ School of Public Health, Zhejiang Chinese Medical University, Hangzhou, China; ^2^ School of Life Science, Zhejiang Chinese Medical University, Hangzhou, China; ^3^ Molecular Medicine Institute, Zhejiang Chinese Medical University, Hangzhou, China; ^4^ Academy of Chinese Medical Sciences, Zhejiang Chinese Medical University, Hangzhou, China; ^5^ Department of Dermatology, Zhejiang Provincial People’s Hospital, People’s Hospital of Hangzhou Medical College, Hangzhou, China

**Keywords:** long non-coding RNA, alcoholic liver disease, messenger RNA, competitive endogenous RNA,, whole transcriptome sequencing

## Abstract

**Background and Aim:** The worldwide prevalence of alcoholic liver disease (ALD) due to escalating alcohol consumption has presented an unprecedented pressure on human health. A few studies have determined long non-coding RNAs (lncRNAs) involved in the pathogenesis of liver diseases. However, the roles of lncRNAs in ALD development is still poorly understood.

**Methods:** An ALD mouse model was established and confirmed. Expression profiles of lncRNAs were obtained by whole transcriptome sequencing. The altered lncRNAs in ALD mice were further verified by qRT-PCR. Gene Ontology (GO) and Kyoto Encyclopedia of Genes and Genomes (KEGG) enrichment analyses were used to enrich the functions of these lncRNAs. In combination with miRNA and mRNA profiles, we constructed concise endogenous RNA (ceRNA) networks. The function of the most up/downregulated lnRNA was further verified and investigated in both ALD model and AML-12 cells.

**Results:** Totally, five downregulated lncRNAs were obtained and verified in ALD mice. The GO term and KEGG pathway analyses revealed that the identified lncRNAs were associated with alcohol-induced hepatic oxidative damage, cellular inflammation, and lipid metabolism. Combination the differentially modulated miRNAs and mRNAs with ceRNA network analysis, we constructed five ceRNA networks and obtained 30 miRNAs and 25 mRNAs that may participate in ALD. Further, we verified and investigate the function of the most downregulated lnc_1700023H06Rik. Depletion lnc_1700023H06Rik reduced genes encoding for lipid metabolism, especially mRNA Acat2 (ENSMUST00000159697) and Pgrmc2 (ENSMUST00000058578) both *in vivo* and *in vitro*. Knocking down lnc_1700023H06Rik induced triglyceride accumulation and lactate dehydrogenase leakage in AML12 cells, consisting with that in alcohol-treated cells.

**Conclusion:** The five remarkably downregulated lncRNAs in ALD mouse model were identified as novel biomarkers, highlighting the key role of lncRNAs in the development of ALD. The effect of lnc_1700023H06Rik plays a pivotal role in lipid deposition and its pathological pathway in ALD needs further investigation.

## Introduction

Continued and excessive alcohol consumption is exacerbating the worldwide incidence of alcoholic liver disease (ALD) (Rehm et al., 2013), According to the Global Status Report on Alcohol and Health (2018), alcohol consumption caused approximately 3 million deaths and nearly half can be attributed to ALD (Lv et al., 2020; Shield et al., 2020). The pathological progression of ALD comprises a spectrum of diseases from liver steatosis and hepatitis to severe fibrosis or even cirrhosis (Liangpunsakul et al., 2016). Although ALD is well-characterized, it cannot be halted or reversed because the underlying mechanisms for ALD initiation or progression are still unclear.

In the past few decades, the proposed mechanisms for ALD have mainly focused on protein-coding genes of alcohol metabolism, reactive oxygen species, formation and inflammation responses (Beier and Arteel, 2010). Technologies such as RNA sequencing and microarray have revealed that non-coding RNAs (ncRNAs) compring up to 80% of the “transcriptional noise,” and thus, also participated in ALD evelopment. However, the role of lncRNAs in ALD is still inconclusive as compared to that of the well-studied endogenous miRNA.

Long non-coding RNAs (lncRNAs, with lengths exceeding 200 nucleotides) are transcribed from various genomic regions, including introns and exons. The competitive endogenous RNA (ceRNA) regulates target gene expression through chromatin remodeling, transcription, and post-transcriptional processing (Zhang et al., 2016; Zhao et al., 2018). They play a critical role in various biological functions and disease processes (Peng et al., 2017). Recently, lncRNAs has been reported to involve in alcohol-related diseases. For instance, serum levels of AK054921 and AK128652 were downregulated in patients with alcohol-cirrhosis, and they were inversely correlated with the survival of these patients, indicating the pathological role in alcoholic cirrhosis (Yang et al., 2017). The lncRNAs NEAT1, Gm5091, and MEG3 participated in alcohol-induced hepatic steatosis and fibrosis in animal models (Wang et al., 2018; Zhou et al., 2018; Ye et al., 2020). Despite the definite role of lncRNAs in alcoholic diseases, the profiles of lncRNAs in ALD models have not been comprehensively investigated.

This study objective to screen the potential lncRNAs that may involved in the development of ALD in mice. We performed RNA sequencing to identify the differentially expressed lncRNAs in liver. The functions of lncRNAs were analyzed using Gene Ontology (GO) and Kyoto Encyclopedia of Genes and Genomes (KEGG) pathways. Several differentially expressed lncRNAs were further verified. A ceRNA network analysis was constructed to understand the lncRNA-miRNA-mRNA crosstalk. In addition to the miRNA sequencing assay, we proposed several key lncRNA pathways that may participate in the development of ALD. Our results may provide new insights into the underlying mechanisms of ALD.

## Materials and Methods

### Animal Experiments

Male C57BL/6J mice weighing 25 ± 0.5 g (mean ± SD) were maintained at the Animal Center of Zhejiang Chinese Medical University. The ALD mouse model was established as previously described (Ding et al., 2017). Mice were divided into two groups of six animals each. The control groups, pair-fed (PF), were fed with an isocaloric control liquid diet (Bioserv, Frenchtown, NJ) and the treated groups, alcohol-fed (AF), were treated with Lieber-DeCarli diet (Bioserv, Frenchtown, NJ) for 5 weeks. In the first 3 days, mice were fed with Lieber-DeCarli diet without alcohol. At the following 2 days, the caloric content of alcohol in the diet was set at 5.5%, and increased to 11% on the 6th and 7th day, 22% at the 2nd week, 27% at the 3rd week, and maintained at 32% at the last 2 weeks. Animals were allowed ad libitum access to diet and water. Mice were euthanized using an intravenous injection of pentobarbital sodium (Merck, Darmstadt, Germany) without any pain after 12 h fasting.

### Cell Culture and Treatment

The alpha mouse liver (AML)-12 hepatocyte cell line was obtained from the American Type Culture Collection (ATCC, Manassas, VA) and cultured in Dulbecco’s Modified Eagle Medium/Ham’s Nutrient Mixture F-12, 1:1 (Sigma, Aldrich, MO), containing 10% (v/v) fetal bovine serum (Life technologies), 5 mg/ml insulin (Sigma), 5 μg/ml transferrin (Sigma), 5 ng/ml selenium (Sigma), 40 ng/ml dexamethasone (Sigma), 100 U/mL penicillin, and 100 μg/ml streptomycin (Life technologies, 15,140–122) at 37°C in a humidified O_2_/CO_2_ (95:5) atmosphere (Li et al., 2014). Cells were plated into 12-well plates, and 200 μmol/L of ethanol was added when the cells volume reached about 80% confluence. After 12 h treatment, cells were collected for qRT-PCR.

### RNA Interference

Based on the results of qRT-PCR, we selected the lncRNA with the most significant differential expression to investigate its function in AML12 cells. Cultured cells were transfected with small interfering RNA (siRNA) against mouse lnc_1700023H06Rik (Ribobio, Guangzhou, China) using Lipofectamine 2000 (Invitrogen, Carlsbad, CA) according to the manufacturer’s instructions. In the control group (NC), cells were transfected with scrambled siRNA (Ribobio). Cells were collected after 24–48 h for qRT-PCR. Sequences of siRNA against mouse lnc_1700023H06Rik were listed in Supplementary Table S1, the sequences of scrambled siRNA were not disclosed.

To investigate the function of lnc_1700023H06Rik in the setting of alcohol treating. Cells were transfected with siRNAs as described aforementioned. Then, 200 μmol/L of ethanol was added into each well. After 12 h, the culture supernatants were for detection of Lactate dehydrogenase (LDH) and the cells were collected for triglyceride (TG) detection.

### Histological and Biochemical Assays

Small pieces of liver were isolated and fixed immediately with 4% paraformaldehyde (Biosharp, Guangzhou, China). Samples were stained with hematoxylin (Sigma) and eosin (Sigma) to determine the histological change. Oil red O (Sigma) staining was performed on fresh liver samples to detect the lipid depostion according to our previous study (Ma et al., 2019). The amounts of liver cholesterol (TC), TG, plasma alanine aminotransferase (ALT), and aspartate aminotransferase (AST) were determined according to the manufacturer’s instructions (Jiancheng, Nanjing, China).

### Whole Transcriptome Sequencing

Total RNA was extracted, and quantified using Bioanalyzer 2100 (Agilent, CA, United States) with RNA Integrity Number (RIN) > 7.0. Subsequently, a small RNA library and a chain-specific library without ribosomal RNA were established and sequenced. The ribosome-deficient chain-specific library obtain the sequence information of lncRNA but also the sequence information of mRNA and circRNA. The small RNA library provided a miRNA sequence.

Transcripts that overlapped with known mRNAs and those that were shorter than 200 bp were discarded. The coding potential of transcripts was determined using Coding Potential Calculator (CPC) (Kong et al., 2007), Coding-Non-Coding Index (CNCI) (Sun et al., 2013), and Pfam (Punta et al., 2012). Transcripts with CPC scores <−1 and CNCI scores <0 was removed. StringTie and fragments per kilobase of transcript per million fragments (FPKM) (Pertea et al., 2015) were used to determine the levels of mRNAs and lncRNAs, respectively. LncRNAs were analyzed using the Ballgown R package. Those with log_2_ fold change (FC) > 1 or log_2_ FC <−1 and *p* value <0.05 were considered differentially expressed (Frazee et al., 2015).

### Validation of Key lncRNAs and mRNA Using qRT-PCR

Total RNA was extracted using TRIzol (Invitrogen, Carlsbad, CA). The content and purity was determinated by, NanoDrop ND-1000 (NanoDrop, Wilmington, DE). The reaction systems containing cDNA templates, SYBR Green (Bimake, Houston, TX), ddH_2_O (Bimake), and primers were mixed for qRT-PCR on ABI 7300 PCR instrument (Thermo Fisher Scientific, Waltha, MA). The gradient temperatures were set at 95°C for 5 min for Hot-Start DNA Polymer; 95°C for 15 s, 60°C for 1 min (40 cycles) for PCR; 95°C for 15 s, 60°C for 1 min, and 95°C for 15 s for melt curve. The relative gene expression was calculated using the 2^−ΔΔCt^ method, and it was normalized to the control group. All primer sequences were listed in Supplementary Table S2-S3.

### Gene Ontology Enrichment Analysis

Gene Ontology functional enrichment analysis maps and calculates the numbers of all the differentially expressed genes to each term in the GO database, Then, the enrichment fractions and the number of differentially expressed genes (S gene number) enriched in each term were determined according to *p* values. Finally, the top 10 terms with *p* < 0.05 and largest numbers of S gene number were analyzed.

### Kyoto Encyclopedia of Genes and Genomes Enrichment Analysis

Biological functions of differentially expressed lncRNAs were determined by KEGG (www.genome.jp/kegg), a public database for genome deciphering. The basic threshold for significant enrichment was set at *p* < 0.05. Subsequently, each pathway was sorted in a descending order according to S gene numbers, and the top ten pathways were displayed.

### ceRNA Network Construction

After verification, lncRNAs with absolute values of log_2_ FC > 2 and *p* < 0.05 were selected. Combination with the differentially expressed miRNA and mRNA in sequence analysis we constructed the ceRNA networks. The RNAs which didn’t obeyed the rule of negative regulation between lncRNA and miRNA, miRNA and mRNA, were removed. Finally, ceRNA network diagrams were prepared using Cytoscape (v3.8.2, http://www.cytoscape.org/) (Liu et al., 2019).

### Detection of Triglyceride and Lactate Dehydrogenase Leakage

The supernatant of AML12 was collected and the activity of LDH leakage was detected according to the instructions of LDH assay kit (Beyotime, Shanghai, China). Cells were then washed with PBS and the triglyceride content in each group was measured according to the instructions of TG assay kit (Jiancheng, Nanjing, China).

### Statistical Analysis

Statistical analyses were carried out using the Student’s *t*-test and ANOVA-test accordingly via the SPSS 25.0 software (Chicago, IL, United States). Significant level was set at *p* < 0.05. The bar charts were prepared using GraphPad Prism 7.0 (GraphPad Software, California, United States).

## Results

### Hepatic Lipid Accumulation and Hepatic Injury Under Alcohol Feeding

**FIGURE 1:**
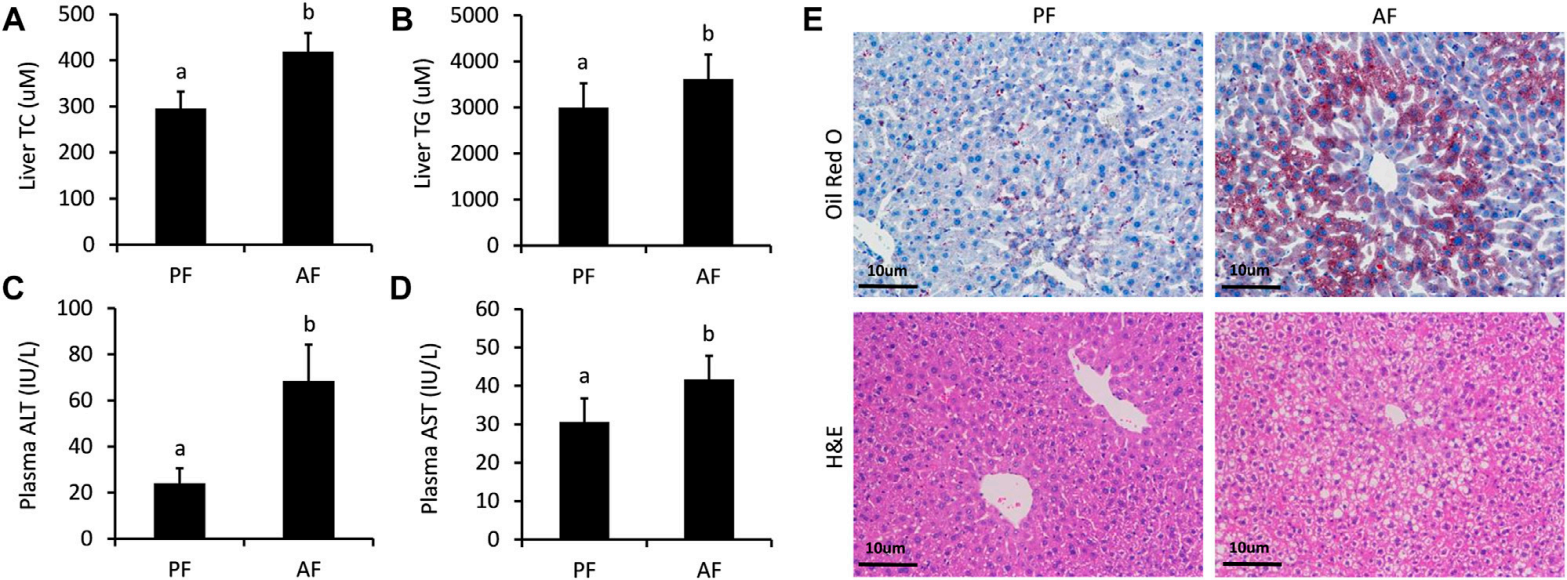
Serum biochemical indices and liver morphologies of ALD mice. **(A)** Hepatic cholesterol (TC) contents. **(B)** Hepatic triglyceride (TG) contents. **(C)** Plasma alanine aminotransferase (ALT) levels. **(D)** Serum aspartate aminotransferase (AST) levels **(E)** Hematoxylin and eosin staining and Oil Red O staining of liver tissues. Bars represents 10 µm. Data are expressed as the mean ± SD (*n* = 6 mice per group).

The successful establishment of ALD mouse model was verified by a sequence of well-characterized endpoints. As described, the amounts of TC (Figure 1A), TG (Figure 1B), ALT (Figure 1C), and AST (Figure 1D) remarkably increased in the AF group as compared to those in the PF group, indicating liver injury and hyperlipidemia. Additionally, a moderate amount of lipid droplets and obvious lipid vacuoles were observed in the liver (Figure 1E). The pathological morphology of the liver pointed toward hepatic steatosis and hepatitis, implicating a well-established ALD mouse model.

### Differentially Expressed lncRNAs Identified in Alcoholic Liver Disease Mice

**FIGURE 2:**
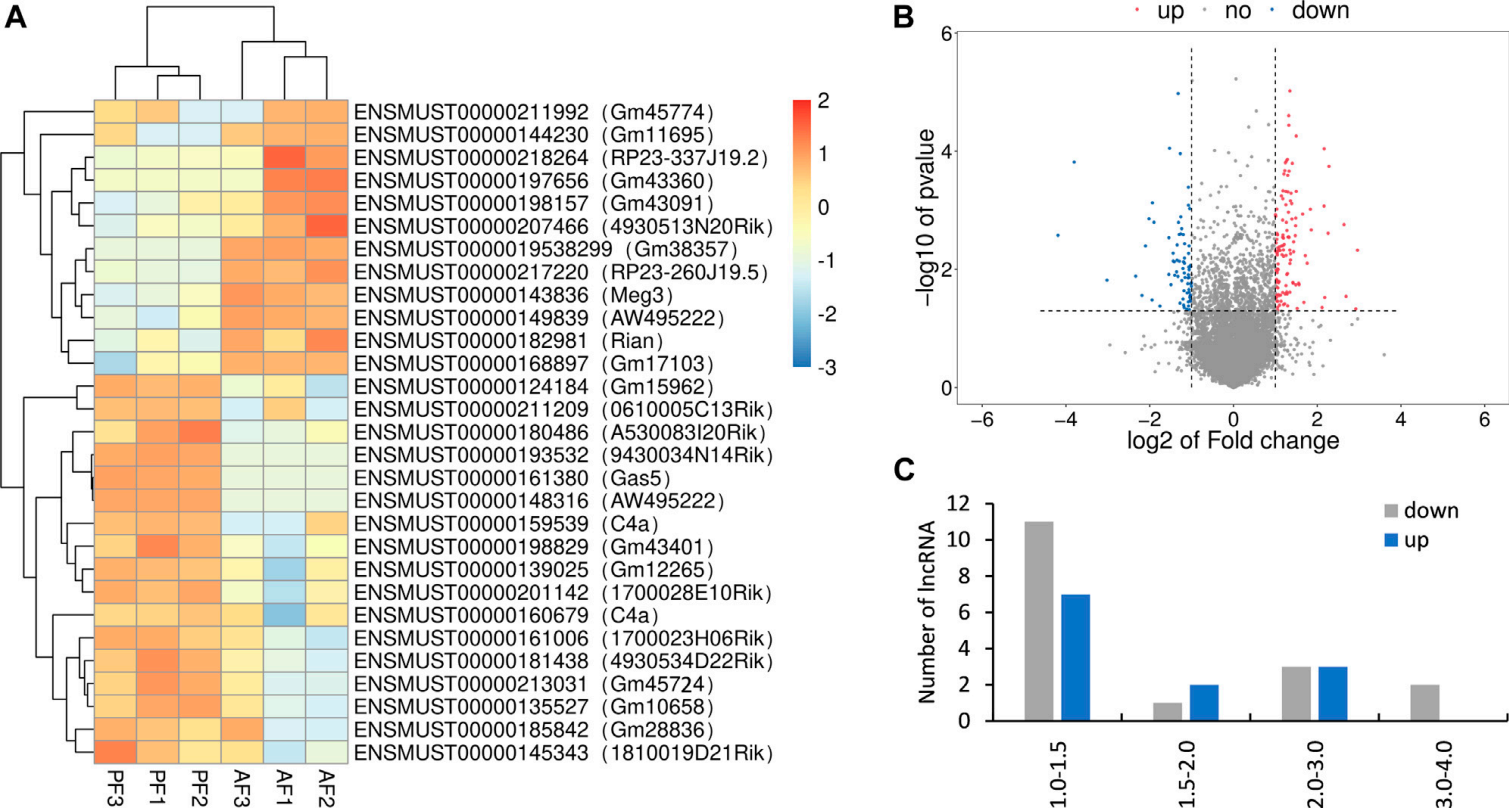
Overview of the RNA sequencing signatures. **(A)** Volcanic map of differentially expressed lncRNAs. **(B)** Hierarchical clustering of the differentially expressed lncRNAs. **(C)** Categories of the 29 differentially expressed lncRNAs.

Totally, twenty-nine differentially expressed lncRNAs were identified by using RNA sequencing. We divided them into two groups: twelve upregulated and Seventeen downregulated, as separated and clustered by volcano graph and heat map (Figure 2A,B). In summary, eighteen lncRNAs with eleven upregulated and seven downregulated genes changed nearly by 2 to 2.8 fold. Three lncRNAs (one upregulated and two downregulated) altered by 2.8 to 4-fold. Six lncRNAs, three upregulated and three downregulated, changed by 4 to 8-fold. The remaining two lncRNAs changed nearly by 8 to 16-fold (Figure 2C). Finally, eight lncRNAs with log_2_ FC change >2.0 (4-fold) and *p* < 0.05 were sent for PCR verification (Table 1).

**TABLE 1:** Top 8 differently expressed lncRNAs in ALD mice model.

lncRNA	*p*-value	Log_2_ of (FC)	Regulation	t_ID	chorm	Strand
mou_lnc_AW495222 (39807)	＜0.01	−3.8040	down	39807	Chr13	—
mou_lnc_AW495222 (39805)	＜0.01	2.6394	up	39805	Chr13	—
mou_lnc_1700023H06Rik	＜0.01	−2.1013	down	39407	chr13	—
mou_lnc_Gm38357	＜0.01	2.9613	up	84884	chr3	—
mou_lnc_0610005C13Rik	0.0276	−2.1850	down	128458	Chr7	—
mou_lnc_Gm12265	0.0131	−2.3356	down	23153	Chr11	—
mou_lnc_Gm45724	0.0153	−3.0207	down	143568	Chr8	—
mou_lnc_Rian	0.0442	2.1151	up	35221	Ch12	—

### Verification 5 Differentially Expressed lncRNAs via qRT-PCR

**FIGURE 3:**
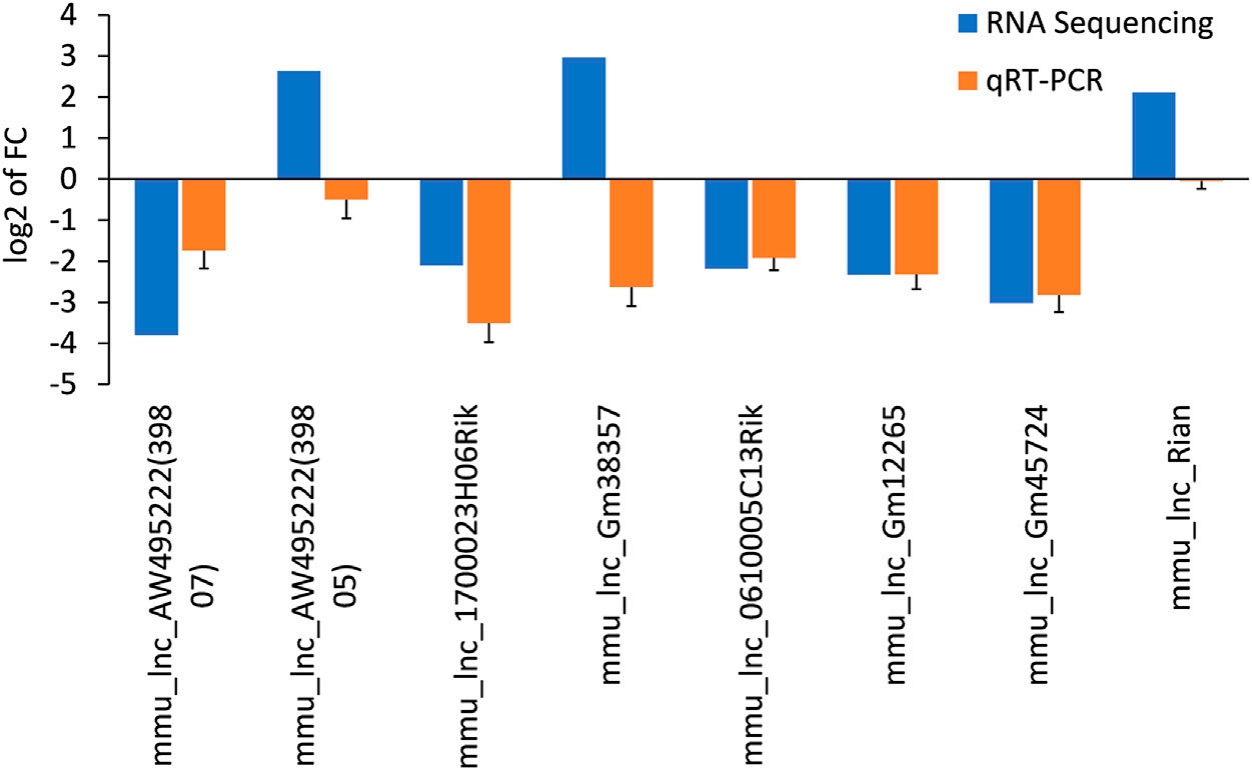
Comparison of the expression levels of twelve lncRNAs between RNA sequencing and qRT-PCR. Data are displayed as log_2_ transformed fold changes (log_2_ FC), and the negative value indicates the downregulated lncRNAs in the AF group, the level of bar column indicates the differential expression multiple of target lncRNA in PF and AF groups (*p*＜0.05).

The differentially expressed lncRNAs were further verified by RT-PCR. Five of the selected lncRNAs in the AF group were maintained as the PF group. Among which, five (mmu_lnc_AW495222(39,807), mmu_lnc_1700023H06Rik, mmu_lnc_0610005C13Rik, mmu_lnc_Gm12265, and mmu_lnc_Gm45724) were consistent with the sequencing results, and two were reversed (mmu_lnc_Gm38357 and mmu_lnc_AW495222(39,805)) (Figure 3). Notably, mou_lnc_AW495222(39,807) and mou_lnc_Gm45724 demonstrated maximum inhibition both in RNA sequencing and PCR verification assays. The expression of mmu_lnc_Rian was not different in PF and AF groups. Altogether, about 5 lncRNAs were verified in RT-PCR.

### Functional Enrichment of Differentially Expressed Genes

**FIGURE 4:**
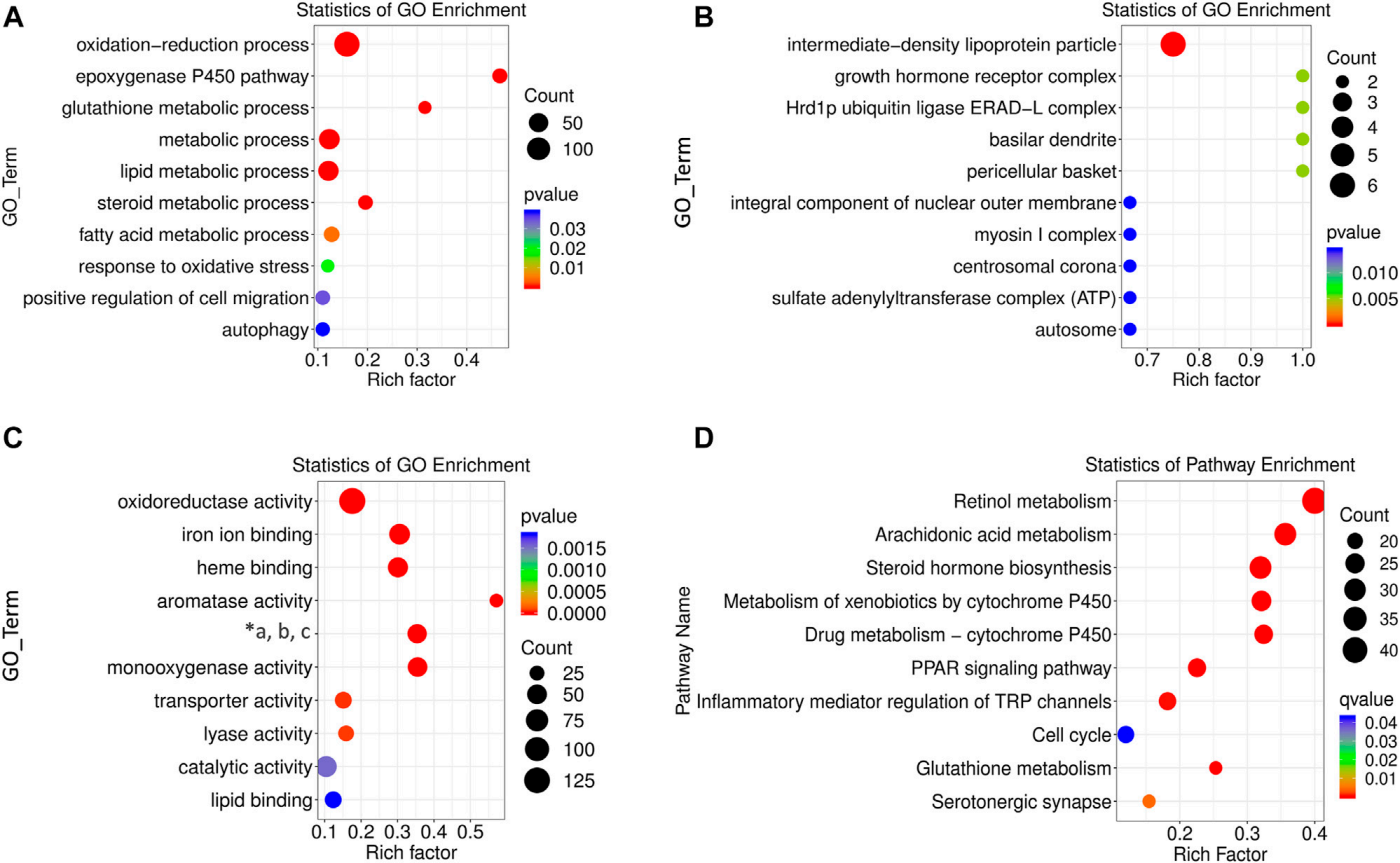
GO and KEGG enrichment analysis of differentially expressed lncRNAs. BP **(A)**, CC **(B)**, and MF **(C)** terms for enrichment (rich factor) of differentially expressed lncRNAs in GO analysis (“*a, b, c” refers to “oxidoreductase activity, acting on paired donors, and molecular oxygen, respectively.”) **(D)** KEGG pathways enriched by differentially expressed lncRNAs. The size of the circle represents S gene numbers. The color depth represents the statistical significance.

Functions of the differentially expressed lncRNAs were separately determined using GO and KEGG enrichment analyses. The first ten pathways enriched from BP, CC, and MF terms were selected using GO analysis. The enriched BP term (Figure 4A) included metabolic processes of lipids, fatty acids, steroid hormones, and oxidation-related pathways with rich factors higher than 0.1. The epoxygenase P450 pathway was ranked the first rich factor in the BP term. All the enriched CC terms had a high rich factor (>0.6) with the most significantly enriched pathway as intermediate density lipoprotein particle (Figure 4B). The MF terms were enriched in oxidative metabolism enzymes, aromatase, and lyase activity as well as iron and heme binding (Figure 4C). Specifically, the rich factor of aromatase activity peaked near 0.6.

The potential pathogenesis of ALD was determined using KEGG enrichment analysis with a rich factor higher than 0.1. In total, ten pathways were enriched, seven of which had rich factors higher than 0.2. They were ranked as retinol metabolism, arachidonic acid metabolism, steroid hormone biosynthesis, metabolism of xenobiotics by cytochrome P450 (CYP450), drug metabolism by CYP450, peroxisome proliferators activated receptor (PPAR) signaling pathway, and glutathione metabolism (Figure 4D).

The ceRNA interaction network of 5 differentially expressed lncRNAs.

**FIGURE 5:**
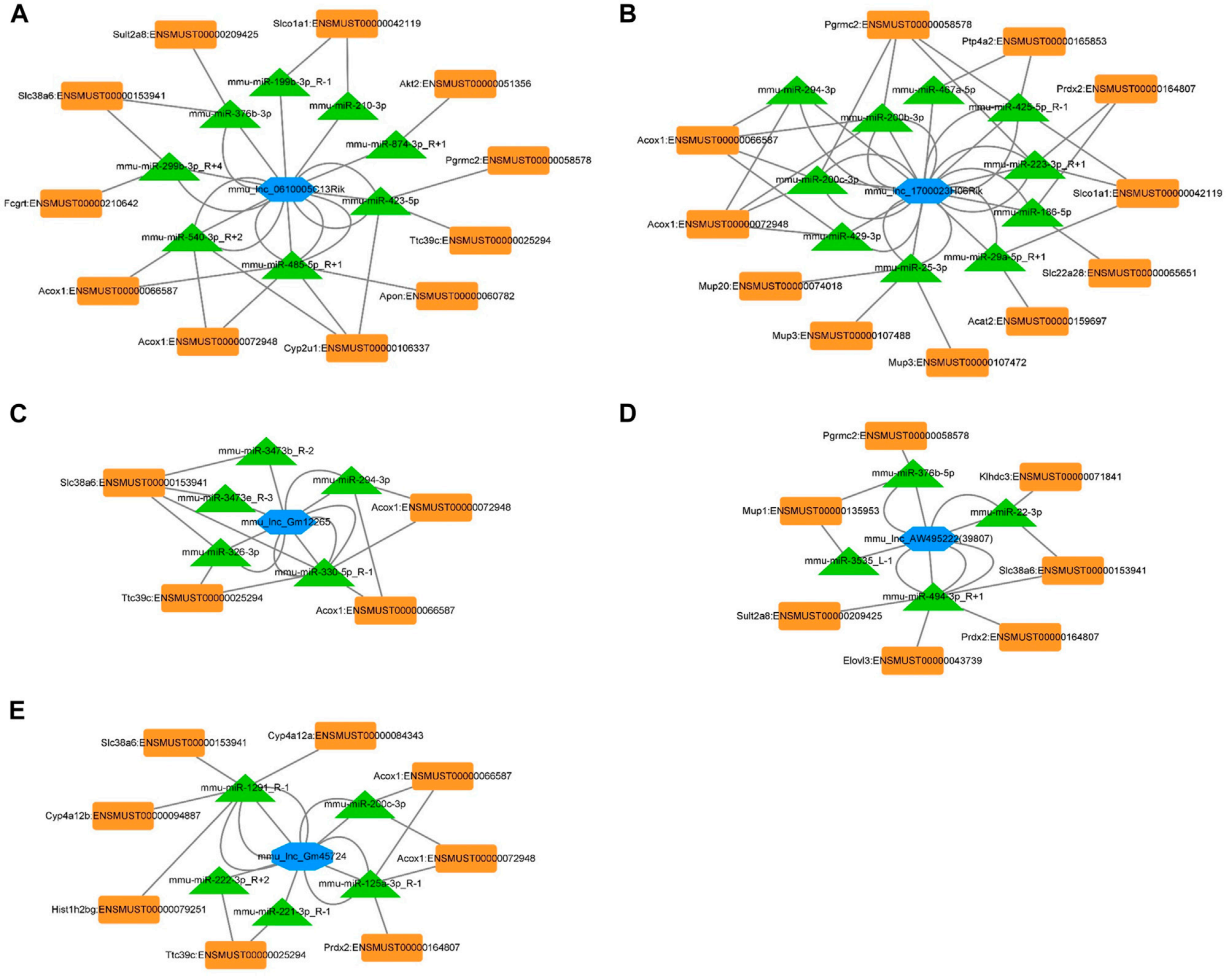
ceRNA networks of mou_lnc_0610005C13Rik, mou_lnc_1700023H06Rik, mou _ lnc _ Gm12265, mou _ lnc _ AW495222(39,807), and mou_lnc_Gm45724. Blue hexagon represents the five lncRNAs. The green triangles and orange quadrilaterals represent differentially expressed miRNA and mRNA, respectively.

The ceRNA network represents a novel regulatory mechanism among lncRNA, miRNA, and mRNA. We chose the top five significantly downregulated lncRNAs (mou_lnc_0610005C13Rik, mou_lnc_1700023H06Rik, mou _ lnc _ Gm12265, mou _ lnc _ AW495222(39,807), and mou_lnc_Gm45724) to construct the ceRNA network. The complex networks were further modified according to our whole transcriptome sequencing analysis data comprising thirty upregulated miRNAs and twenty-five downregulated mRNAs (Supplementary Table S4). Finally, the five aforementioned lncRNAs targeted eight miRNAs and eleven mRNAs (Figure 5A), ten miRNAs and eleven mRNAs (Figure 5B), five miRNAs and four mRNAs (Figure 5C), four miRNAs and seven mRNAs (Figure 5D), and five miRNAs and eight mRNAs (Figure 5E), respectively.

Verification on lnc_1700023H06Rik ceRNA network in both *in vivo* and *in vitro* model.

**FIGURE 6:**
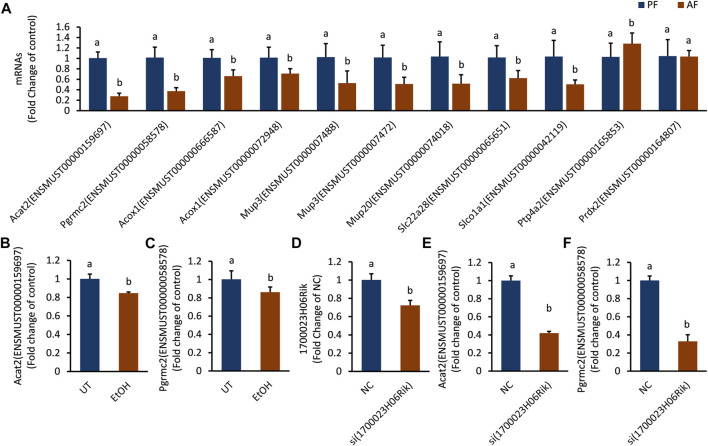
The expression trend of mRNAs corresponding to lnc_1700023H06Rik. **(A)** The expression trends of 11 mRNAs in mouse liver tissues. **(B,C)** Expression of Acat2, Pgrmc2 in ethanol-treated AML-12 cells. **(D)** 1700023H06Rik expression in AML-12 cells by siRNA. **(E,F)** Expression of Acat2 and Pgrmc2 after knocking down lnc_1700023H06Rik.

The mRNAs linked to the most downregulated lncRNA (lnc_1700023H06Rik) were verified in mouse liver and *in vitro* hepatocyte by RT-PCR. The expressions of Acat2 (ENSMUST00000159697), Pgrmc2 (ENSMUST00000058578), Acox1 (ENSMUST000000666587), Acox1 (ENSMUST00000072948), Mup3 (ENSMUST0000007488), Mup3 (ENSMUST 0000007472), Mup20 (ENSMUST00000074018), Slc22a28 (ENSMUST00000065651) and Slco1a1 (ENSMUST00000042119) were down-regulated in the AF group, which was consistent with the sequencing results (Supplementary Table S4). The mRNA level of Ptp4a2 (ENSMUST 00000165853) was upregulated and Prdx2 (ENSMUST00000164807) was not changed in AF groups (Figure 6A). The transcription level of Acat2 and Pgrmc2 was further corroborated in ethanol-treated AML-12 cells (Figure 6B,C). Knocking down lnc_1700023H06Rik (Figure 6D) revealed downregulation of both Acat2 and Pgrmc2 in AML-12 cells (Figure 6E,F), which was consistent with that in mice model.

### Interference lnc_1700023H06Rik Resulted in Triglyceride Accumulation and Cell Damage

**FIGURE 7:**
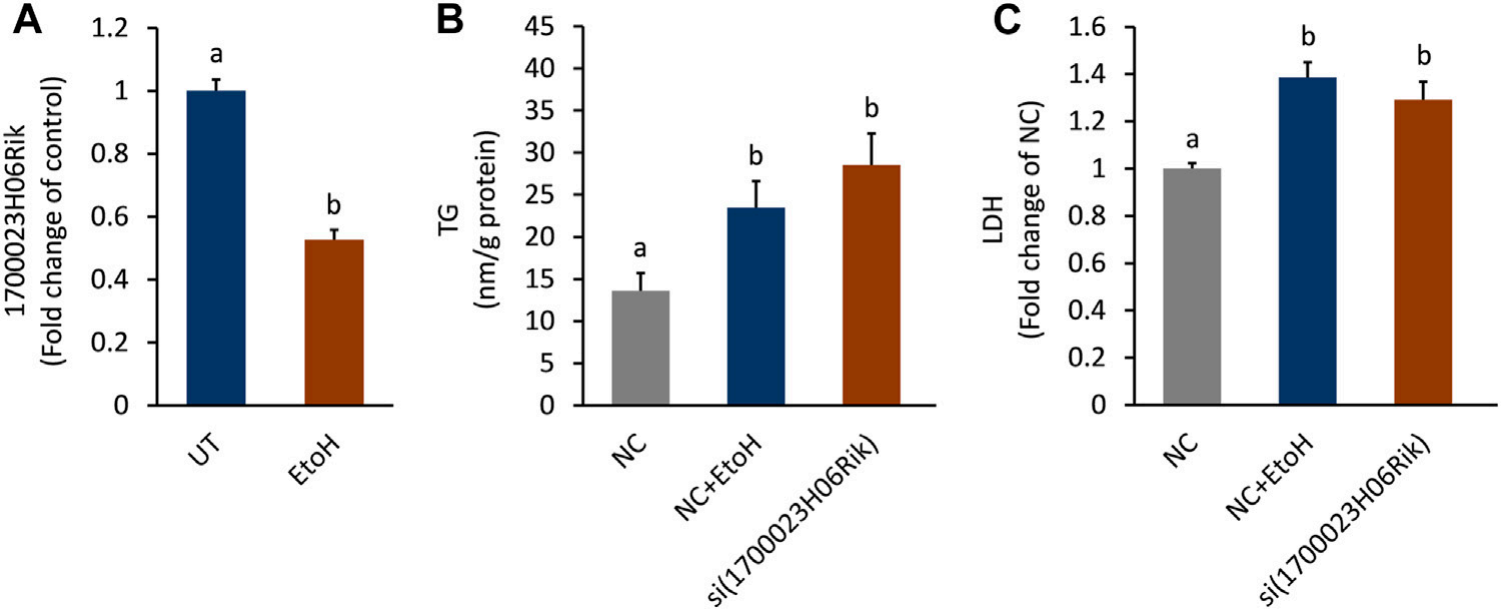
Effect of lnc_1700023H06Rik on triglyceride accumulation and cell damage. **(A)** Expression of lnc_1700023H06Rik in ethanol-treated AML-12 cells. **(B)** TG accumulation in AML-12 cells after knocking down lnc_1700023H06Rik. **(C)** LDH leakage in AML-12 cells after knocking down lnc_1700023H06Rik.

The biological function of lnc_170023H06Rik was further investigated in AML-12 cells. In line with the PCR and RNA-seq analysis, lnc_170023H06Rik was down-regulated in AML-12 cells after exposure to alcohol (Figure 7A). Depletion lnc_170023H06Rik was accompanied with apparent TG accumulation and LDH leakage in AML12 cells, which was consistent with that of ethanol treatment (Figure 7B,C).

## Discussion

In recent decades, many studies have been conducted to determine the pathology of ALD. Recently, the cardinal role of lncRNAs in ALD has been recognized. A few lncRNAs have been identified as biomarkers for predicting survival in patients with alcoholic cirrhosis (Yang et al., 2017). For the first time, we prioritized and confirmed eight new lncRNAs that may be involved in the pathological of ALD. Furthermore, we established the top five lncRNA-modulated networks based on the results of whole transcriptome sequencing.

Based on the co-expressed mRNAs functions of differentially expressed lncRNAs could be mainly ascribed to the following categories: oxidative stress, lipid metabolism and inflammatory reactions. According to the KEGG enrichment analysis, we determined the metabolism of xenobiotics by CYP450, drug metabolism by CYP450, retinol metabolism, and arachidonic acid metabolism as the most enriched pathways. Consistent with our present and previous findings, alcohol intake triggers RNAs involved in xenobiotic and drug biodegradation pathways (Dou et al., 2020). CYP450 is crucial for chemical metabolism. Alcohol-related hepatic oxidative stress is caused by ethanol oxidation by its superfamily CYP2E1, which ultimately promotes the development of ALD (Lu et al., 2018). Normally, the liver would exert anti-oxidative and anti-inflammatory processes by converting arachidonic acid into biologically active cyclic trienoic acid through arachidonic acid epoxygenase (Wells et al., 2016). However, CYP450 activation induces arachidonic acid hydroxylation and epoxidation, which partially lead to oxidative stress and liver peroxidation after alcohol intake (Seitz and Mueller, 2019). Additionally, alcohol can cause the decrease of retinol content in liver, and activation CYP2E1 is the main reason for this effect (Clugston et al., 2015). Results from the KEGG analysis were consistent with those of the GO analysis, as the most enriched CC, BF, and MF terms were occupied by lipid and alcoholic metabolism.

Although the functions of lncRNAs have not been fully recognized, the interacting with miRNAs and mRNAs from the ceRNA network enable the understanding of the pathological roles of lncRNAs in ALD. As mentioned above, the initial pathological feature of ALD is hepatic steatosis, that is, ALD is closely related to lipid metabolism. This study found that lnc_0610005c13Rik-miR540, lnc_Gm45724-miR200c-3p, lnc_1700023H06Rik-miR294-3p, miR200c-3p, miR200b-3p, lnc_12,265-miR294-3p, and -miR 330-5p regulated the mRNA of Acox1, the first enzyme in fatty acid oxidation. Moreover, induction of miR540 inhibited the proteins involved in fatty acid oxidation, such as Acox1, PPAR⍺, and cpt1⍺, in ALR-L-knockout mice (Kumar et al., 2019). And the expression of Acox1 was down-regulated in alcohol-treated mice with increased lipid accumulation in the mouse liver (Sun et al., 2016). Thus, mitigating these lncRNAs remarkably blocked the activities of enzymes involved in fatty acid oxidation, leading to lipid accumulation. These predictions were further verified in lnc_1700023H06Rik. Both lnc_1700023H06Rik and its related mRNAs, especially Acat2 and Pgrmc2 were downregulated in ALD mice and alcohol-treated cells. Genetic depletion of Acat2 and Pgrmc2 leads to excessive deposition of free fatty acid *in vivo*, and lipid metabolic dysfunction (Wang et al., 2017; Galmozzi et al., 2019). Ectopic Lipid accumulation and the consequence of lipotoxicity is the hallmark for liver injury in alcohol feeding mice (Alpini, 2019). Our study *in vitro* confirmed the blunted lnc_1700023H06Rik tightly contributed to lipid accumulation and cell injury when response to alcohol treatment.

Besides lipid deposition, oxidative stress and inflammation are two pathological factors in ALD. The mRNAs including Prdx2, slc38a6, and sult2a8, engaging in the network of lnc_1700023H06Rik-miR223-3p and lnc_AW49522-miR494-3p are responsible for the anti-oxidative and glutamate transportation pathways (Chan et al., 2016; Shimohira et al., 2018; Wang et al., 2020). Diminishing Prdx2 in hepatocyte of alcohol-treated mice endows lipid deposition (Han et al., 2020). Thus, attenuation of both lnc_1700023H06Rik and lnc_AW49522 in ALD mice indicated the deterioration of anti-oxidative system and lipid metabolism. Additionally, downregulation of lnc_Gm12265, lnc_Gm45724 and lnc_1700023H06Rik may involve in inflammation signaling. Activation of miR326 by inhibition the expression of lnc_Gm12265 may promoted hepatic inflammation as miR326 participates in the secretion of inflammation cytokines via TLR4/myD88/NFκB signaling (Liao et al., 2019). In contrast, miR223-3p, an emerging negative regulator of NLRP3 inflammation and inactivator of hematopoietic stem cells (Jimenez Calvente et al., 2020), was also triggered in the ALD mouse model through the downregulation of lnc_Gm45724 or lnc_1700023H06Rik. Thus, we posited that miR223-3p may participated in ALD given that NLRP3 is responsible for innate immunity during acute liver injury (Huang et al., 2013). The mRNA of Akt2, engaging in the network of lnc_0610005C13Rik, may also contribute to the inflammation status in ALD mice since inhibition on Akt2 attenuated the transcription of inflammation cytokine under alcohol challenge (Reyes-Gordillo et al., 2019).

The limitation of this study lies in the low sequence alignment between mice lncRNAs and the whole human genome via the sequence homological alignment. Most of lncRNAs that identified by our studies cannot be successfully paired in the sequences of humans. Only mmu _ lnc _ 170023H06Rik has a high homology between mice and human (ARRDC3-AS1, 89.58%, 600 bases, Supplementary Table S5). About 20 to 30 bases of mmu_lnc_Rian in mice has a full homology with human lncRNAs (LINC02315, LINC02307). Unexpectedly, none of the compared three human lncRNAs (ARRDC3-AS1, LINC02315, LINC02307) has been reported to be related to ALD. Despite this limitation, our study still sets the stage for further investigation on ALD development because the pathological mechanism of ALD in human is still in its infant stage.

In conclusion, the present study identified five lncRNAs functioning as anti-inflammation, anti-oxidation, and lipid metabolism in ALD mice model. Combination ceRNA network with miRNAs and mRNAs sequencing data, we got five ceRNA networks. Taken the most downregulated lncRNA as an example, we, for the first time, verified lnc_1700023H06Rik is tightly related to lipotoxicity in ALD mice model. However, the exact activities of the identified lncRNAs and the proposed pathways still need verification. Despite this limitation, our study highlights the identified lncRNAs as key factors responsible for ALD pathogenesis.

## Data Availability

The datasets presented in this study can be found in online repositories. The names of the repository/repositories and accession number(s) can be found below: https://www.ncbi.nlm.nih.gov/geo/, GSE179648; https://www.ncbi.nlm.nih.gov/geo/, GSE175804.

## References

[B1] Alpini G. (2019). Sphingosine Lipid Signaling in Alcoholic Liver Injury. Dig. Liver Dis. 51 (8), 1164–1165. 10.1016/j.dld.2019.04.002 31031176

[B3] Beier J. I. Arteel G. E. (2010). Ethanol-Induced Hepatotoxicity. Compr. toxicologe 9, 421–435. 10.1016/B978-0-12-801238-3.95666-610.1016/b978-0-08-046884-6.01017-4

[B4] Chan K. Busque S. M. Sailer M. Stoeger C. Bröer S. Daniel H. (2016). Loss of Function Mutation of the Slc38a3 Glutamine Transporter Reveals its Critical Role for Amino Acid Metabolism in the Liver, Brain, and Kidney. Pflugers Arch. 468 (2), 213–227. 10.1007/s00424-015-1742-0 26490457

[B5] Clugston R. D. Huang L. S. Blaner W. S. (2015). Chronic Alcohol Consumption Has a Biphasic Effect on Hepatic Retinoid Loss. FASEB J. 29 (9), 3654–3667. 10.1096/fj.14-266296 25985802 PMC4550375

[B6] Ding L. Wo L. Du Z. Tang L. Song Z. Dou X. (2017). Danshen Protects against Early-Stage Alcoholic Liver Disease in Mice via Inducing PPARα Activation and Subsequent 4-HNE Degradation. PloS one 12 (10), e0186357. 10.1371/journal.pone.0186357 29020055 PMC5636149

[B7] Dou X. Feng L. Ying N. Ding Q. Song Q. Jiang F. (2020). RNA Sequencing Reveals a Comprehensive Circular RNA Expression Profile in a Mouse Model of Alcoholic Liver Disease. Alcohol. Clin. Exp. Res. 44 (2), 415–422. 10.1111/acer.14265 31840820

[B8] Frazee A. C. Pertea G. Jaffe A. E. Langmead B. Salzberg S. L. Leek J. T. (2015). Ballgown Bridges the gap between Transcriptome Assembly and Expression Analysis. Nat. Biotechnol. 33 (3), 243–246. 10.1038/nbt.3172 25748911 PMC4792117

[B9] Galmozzi A. Kok B. P. Kim A. S. Montenegro-Burke J. R. Lee J. Y. Spreafico R. (2019). PGRMC2 Is an Intracellular Haem Chaperone Critical for Adipocyte Function. Nature 576 (7785), 138–142. 10.1038/s41586-019-1774-2 31748741 PMC6895438

[B10] Han Y. H. Li W. L. Jin M. H. Jin Y. H. Zhang Y. Q. Kong L. Z. (2020). Peroxiredoxin II Inhibits Alcohol-Induced Apoptosis in L02 Hepatocytes through AKT/β-Catenin Signaling Pathway. Anticancer Res. 40 (8), 4491–4504. 10.21873/anticanres.14454 32727779

[B11] Huang H. Chen H. W. Evankovich J. Yan W. Rosborough B. R. Nace G. W. (2013). Histones Activate the NLRP3 Inflammasome in Kupffer Cells during Sterile Inflammatory Liver Injury. J. Immunol. 191, 2665–2679. 10.4049/jimmunol.1202733 23904166 PMC3777242

[B12] Jimenez Calvente C. Del Pilar H. Tameda M. Johnson C. D. Feldstein A. E. (2020). Del Pilar H.MicroRNA 223 3p Negatively Regulates the NLRP3 Inflammasome in Acute and Chronic Liver Injury. Mol. Ther. 28 (2), 653–663. 10.1016/j.ymthe.2019.09.013 31585800 PMC7000998

[B13] Kong L. Zhang Y. Ye Z.-Q. Liu X.-Q. Zhao S.-Q. Wei L. (2007). CPC: Assess the Protein-Coding Potential of Transcripts Using Sequence Features and Support Vector Machine. Nucleic Acids Res. 35, W345–W349. 10.1093/nar/gkm391 17631615 PMC1933232

[B14] Kumar S. Rani R. Karns R. Gandhi C. R. (2019). Augmenter of Liver Regeneration Protein Deficiency Promotes Hepatic Steatosis by Inducing Oxidative Stress and microRNA-540 Expression. FASEB J. 33 (3), 3825–3840. 10.1096/fj.201802015R 30540918 PMC6404588

[B15] Li S. Li J. Shen C. Zhang X. Sun S. Cho M. (2014). tert-Butylhydroquinone (tBHQ) Protects Hepatocytes against Lipotoxicity via Inducing Autophagy Independently of Nrf2 Activation. Biochim. Biophys. Acta 1841 (1), 22–33. 10.1016/j.bbalip.2013.09.004 24055888 PMC3884638

[B16] Liangpunsakul S. Haber P. McCaughan G. W. (2016). Alcoholic Liver Disease in Asia, Europe, and North America. Gastroenterology 150 (8), 1786–1797. 10.1053/j.gastro.2016.02.043 26924091 PMC4887319

[B17] Liao X. Zhan W. Tian T. Yu L. Li R. Yang Q. (2019). MicroRNA‐326 Attenuates Hepatic Stellate Cell Activation and Liver Fibrosis by Inhibiting TLR4 Signaling. J. Cell Biochem 121, 3794–3803. 10.1002/jcb.29520 31692098

[B18] Liu S. Xie X. Lei H. Zou B. Xie L. (2019). Identification of Key circRNAs/lncRNAs/miRNAs/mRNAs and Pathways in Preeclampsia Using Bioinformatics Analysis. Med. Sci. Monit. 25, 1679–1693. 10.12659/MSM.912801 30833538 PMC6413561

[B19] Lu Y. Cederbaum A. I. (2018). Cytochrome P450s and Alcoholic Liver Disease. Curr. Pharm. Des. 24 (14), 1502–1517. 10.2174/1381612824666180410091511 29637855 PMC6053342

[B20] Lv Y. So K. F. Xiao J. (2020). Liver Regeneration and Alcoholic Liver Disease. Ann. Transl Med. 8 (8), 567. 10.21037/atm.2020.02.168 32775368 PMC7347779

[B21] Ma Y. Chai H. Ding Q. Qian Q. Yan Z. Ding B. (2019). Hepatic SIRT3 Upregulation in Response to Chronic Alcohol Consumption Contributes to Alcoholic Liver Disease in Mice. Front. Physiol. 10, 1042. 10.3389/fphys.2019.01042 31474877 PMC6707764

[B22] Peng W. X. Koirala P. Mo Y. Y. (2017). LncRNA-mediated Regulation of Cell Signaling in Cancer. Oncogene 36 (41), 5661–5667. 10.1038/onc.2017.184 28604750 PMC6450570

[B23] Pertea M. Pertea G. M. Antonescu C. M. Chang T. C. Mendell J. T. Salzberg S. L. (2015). StringTie Enables Improved Reconstruction of a Transcriptome from RNA-Seq Reads. Nat. Biotechnol. 33 (3), 290–295. 10.1038/nbt.3122 25690850 PMC4643835

[B24] Punta M. Coggill P. C. Eberhardt R. Y. Mistry J. Tate J. Boursnell C. (2012). The Pfam Protein Families Database. Nucleic Acids Res. 40, D290–D301. 10.1093/nar/gkr1065 22127870 PMC3245129

[B25] Rehm J. Samokhvalov A. V. Shield K. D. (2013). Global burden of Alcoholic Liver Diseases. J. Hepatol. 59 (1), 160–168. 10.1016/j.jhep.2013.03.007 23511777

[B26] Reyes-Gordillo K. Shah R. Arellanes-Robledo J. Cheng Y. Ibrahim J. Tuma P. L. (2019). Akt1 and Akt2 Isoforms Play Distinct Roles in Regulating the Development of Inflammation and Fibrosis Associated with Alcoholic Liver Disease. Cells 8 (11), 1337. 10.3390/cells8111337 PMC6912497 31671832

[B27] Seitz H. K. Mueller S. (2019). The Role of Cytochrom P4502E1 in Alcoholic Liver Disease and Alcohol Mediated Carcinogenesis. Z. Gastroenterol. 57 (1), 37–45. 10.1055/a-0784-8815 30641601

[B28] Shield K. Manthey J. Rylett M. Probst C. Wettlaufer A. Parry C. D. H. (2020). National, Regional, and Global Burdens of Disease from 2000 to 2016 Attributable to Alcohol Use: a Comparative Risk Assessment Study. Lancet Public Health 5 (1), e51–e61. 10.1016/S2468-2667(19)30231-2 31910980

[B29] Shimohira T. Kurogi K. Liu M. C. Suiko M. Sakakibara Y. (2018). The Critical Role of His48 in Mouse Cytosolic Sulfotransferase SULT2A8 for the 7α-Hydroxyl Sulfation of Bile Acids. Biosci. Biotechnol. Biochem. 82 (8), 1359–1365. 10.1080/09168451.2018.1464897 29685090

[B30] Sun L. Luo H. Bu D. Zhao G. Yu K. Zhang C. (2013). Utilizing Sequence Intrinsic Composition to Classify Protein-Coding and Long Non-coding Transcripts. Nucleic Acids Res. 41 (17), e166. 10.1093/nar/gkt646 23892401 PMC3783192

[B31] Sun Q. Zhang W. Zhong W. Sun X. Zhou Z. (2016). Dietary Fisetin Supplementation Protects against Alcohol-Induced Liver Injury in Mice. Alcohol. Clin. Exp. Res. 40 (10), 2076–2084. 10.1111/acer.13172 27575873 PMC5742867

[B32] Wang Q. Li M. Shen Z. Bu F. Yu H. Pan X. (2018). The Long Non-coding RNA MEG3/miR-Let-7c-5p Axis Regulates Ethanol-Induced Hepatic Steatosis and Apoptosis by Targeting NLRC5. Front. Pharmacol. 9, 302. 10.3389/fphar.2018.00302 29692724 PMC5902529

[B33] Wang S. Chen Z. Zhu S. Lu H. Peng D. Soutto M. (2020). PRDX2 Protects against Oxidative Stress Induced by *H. pylori* and Promotes Resistance to Cisplatin in Gastric Cancer. Redox Biol. 28, 101319. 10.1016/j.redox.2019.101319 31536951 PMC6811995

[B34] Wang Y. J. Bian Y. Luo J. Lu M. Xiong Y. Guo S. Y. (2017). Cholesterol and Fatty Acids Regulate Cysteine Ubiquitylation of ACAT2 through Competitive Oxidation. Nat. Cell Biol 19 (7), 808–819. 10.1038/ncb3551 28604676 PMC5518634

[B35] Wells M. A. Vendrov K. C. Edin M. L. Ferslew B. C. Zha W. Nguyen B. K. (2016). Characterization of the Cytochrome P450 Epoxyeicosanoid Pathway in Non-alcoholic Steatohepatitis. Prostaglandins Other Lipid Mediat 125, 19–29. 10.1016/j.prostaglandins.2016.07.002 27401401 PMC5035202

[B36] Yang Z. Ross R. A. Zhao S. Tu W. Liangpunsakul S. Wang L. (2017). LncRNA AK054921 and AK128652 Are Potential Serum Biomarkers and Predictors of Patient Survival with Alcoholic Cirrhosis. Hepatol. Commun. 1 (6), 513–523. 10.1002/hep4.1061 29104954 PMC5665385

[B37] Ye J. Lin Y. Yu Y. Sun D. (2020). LncRNA NEAT1/microRNA-129-5p/SOCS2 axis Regulates Liver Fibrosis in Alcoholic Steatohepatitis. J. Transl Med. 18 (1), 445. 10.1186/s12967-020-02577-5 33228663 PMC7686721

[B38] Zhang Y. Xu Y. Feng L. Li F. Sun Z. Wu T. (2016). Comprehensive Characterization of lncRNA-mRNA Related ceRNA Network across 12 Major Cancers. Oncotarget 7 (39), 64148–64167. 10.18632/oncotarget.11637 27580177 PMC5325432

[B39] Zhao Y. Wang H. Wu C. Yan M. Wu H. Wang J. (2018). Construction and Investigation of lncRNA-Associated ceRNA Regulatory Network in Papillary Thyroid Cancer. Oncol. Rep. 39 (3), 1197–1206. 10.3892/or.2018.6207 29328463 PMC5802034

[B40] Zhou B. Yuan W. Li X. (2018). LncRNA Gm5091 Alleviates Alcoholic Hepatic Fibrosis by Sponging miR-27b/23b/24 in Mice. Cell Biol Int 42 (10), 1330–1339. 10.1002/cbin.11021 29935035

