# Supplementary material for: Comprehensive Analysis of the Expression Profiles of Hepatic lncRNAs in the Mouse Model of Alcoholic Liver Disease
Source: Front Pharmacol. 2021 Jul 29;12:709287. doi: 10.3389/fphar.2021.709287 (PMC8358650; doi:10.3389/fphar.2021.709287)
Supplement: Supplementary file 1 [file DataSheet1.docx]

**Comprehensive analysis of the expression profiles of hepatic lncRNAs in the mouse model of alcoholic liver disease**

Xiaobing Dou ^1, 2, 3, †^, Wenwen Yang ^1, 2, †^, Qinchao Ding ^2, 4^, Qiang Han ^1, 4^, Qianyu Qian ^2, 3^, Zhongyan Du ^4^, Yibin Fan ^5^, Cui Wang ^2, 3, 4 *^, Songtao Li ^1, 3, 4 *^

^1^ School of Public Health, Zhejiang Chinese Medical University, Hangzhou, 310053, China

^2^ School of Life Science, Zhejiang Chinese Medical University, Hangzhou, 310053, China

^3^ Molecular Medicine Institute, Zhejiang Chinese Medical University, Hangzhou, 310053, China

^4^ Academy of Chinese Medical Sciences, Zhejiang Chinese Medical University, Hangzhou, 310053, China

^5^ Department of Dermatology, Zhejiang provincial people’s hospital, People’s Hospital of Hangzhou Medical College, Hangzhou, 310014, China

*Corresponding authors:

Songtao Li

School of Public Health, Zhejiang Chinese Medical University, Hangzhou, 310053, China, Tel: +86-571-86613746, Fax: +86-571-86613746, E-mail: lisongtao@zcmu.edu.cn

Cui Wang

School of Life Science, Zhejiang Chinese Medical University, Hangzhou, 310053, China, [Tel: +86-571-86613626](mailto:Tel:%20+86-571-86613626), E-male: [wangcui198506@163.com](mailto:wangcui198506@163.com)

† These authors contribute equally to this paper.

**Disclosure statement**

The authors have declared no conflict of interest

**Acknowledgements**

This work was supported by the National Natural Science Foundations of China (No. 81973041, 81773981, and 81773422), Zhejiang Natural Science Foundations for Distinguished Young Scholars (No. LR20H260001), Special Support Program for High Level Talents in Zhejiang Province (No. ZJWR0308092), and Research Foundations of Zhejiang Chinese Medicine University (No.2020ZR07).

**Materials and Methods**

**RNA homology comparison between mouse and human**

The sequence information of mmu_lnc_1700023H06Rik (ENSMUST00000161006), mmu_lnc_Rian (ENSMUST00000182981), mmu_lnc_Gm12265 (ENSMUST00000139025), mmu_lnc_AW495222 (ENSMUST00000148316), and mmu_lnc_Gm45724 (ENSMUST00000213031) was obtained via Ensumbl (<http://asia.ensembl.org/index.html>). These sequences were sent to compare with that in the whole human genome. The locus information in the segment of human genome was retrieved according to the interval information of the target gene, and finally got the comparison results.

**Results**

**Table S1. sequences of siRNA**

| lncRNA | **sequence** |
| --- | --- |
| Lnc_1700023H06Rik | AACGAGAGCGCTGTCATTAG  GCGTGTAACCAATCTGCTGA  TCATTGAATCCACGTTCCAA  CGCTGTCATTAGGCATTGT  CTGAACTCTTCGAGGCAAA  CGTGTAACCAATCTGCTGA |

**Table S2. Primer sequence of lncRNAs**

| lncRNA | Forward | Reverse |
| --- | --- | --- |
| mou_lnc_AW495222（39807） | GAGCCCAGGGAGAGGAAGCAG | ACAGAGAAGCCACCCCTTGAGAG |
| mou_lnc_AW495222（39805） | AACTTGAGTTCGCTGCAGCA | ATCTCAGCGATGATGCATTCAAGTT |
| mou_lnc_1700023H06Rik | GCGTGTAACCAATCTGCTGAGAGG | AGAAGCGGTTCAATCCTGTTTCGG |
| mou_lnc_Gm38357 | CCATTCCCAGGGACAGTTTCA | CTGCAACTGCAGGGGTTAGA |
| mou_lnc_0610005C13Rik | CAAGTCCCCACCGGGATGC | ACTTTGCCCTGCATGGTGGAT |
| mou_lnc_Gm12265 | AGCCAACTTTCCCAAACTGC | CTGTTGGAGGACTCGCTTCT |
| mou_lnc_Gm45724 | TTTCCATCCTTGCTTCCCTGTG | CTCTGACTGGCTGTCCTGCTA |
| mou_lnc_Rian | CTGGCAAGGACACTTTCTACTC | AATGTCCTCGGCTGCATAGAAG |

**Table S3. Primer sequence of mRNAs**

| mRNA | Forward | Reverse |
| --- | --- | --- |
| actin | GGCTGTATTCCCCTCCATCG | CCAGTTGGTAACAATGCCATGT |
| Prdx2 | CTTCGCCAGATCACAGTCAA | CCTTGCTGTCATCCACATTG |
| Acat2 | CTGTCACAGAACAGGGCAGA | TGACAGTTCCTGTCCCATCA |
| Acox1(66578) | TTATGCGCAGACAGAGATGG | GGCATGTAACCCGTAGCACT |
| Acox1(72948) | CCACATATGACCCCAAGACC | AGGCATGTAACCCGTAGCAC |
| Pgrmc2 | TTCTGCCTGGATAAGGATGC | TGATCCTTGGTGTCCTCCTC |
| Mup3(107472) | ATGACTGCGATTGGTGAACA | ATTCCATGCTCCTCGCATAG |
| Mup3(084531) | ATGACTGCGATTGGTGAACA | ATTCCATGCTCCTCGCATAG |
| Mup20 | GAGCTCTATGGCCGAGAACC | GCAGCGATTGGCATTGGTTA |
| Slco1a1 | TCTTGCCCAAATACCTGGAG | GGACAGGCCAAATGCTATGT |
| Slc22a28 | ATTGTGGTCACATTGGCAGC | GACTCCCATACCTGTTGCCC |
| Ptp4a2 | TGTGTTGCAGTGCATTGTGT | GCGTAACCGCATCTTAGGTC |

**Table S4. Differential expression of miRNAs and mRNAs in ALD mice model and CeNetwork**

| **miR_name** | **up/down** | **mR_name** | **up/down** |
| --- | --- | --- | --- |
| mmu-miR-299b-3p_R+4 | up | Slco1a1:ENSMUST00000042119 | down |
| mmu-miR-199b-3p_R-1 | up | Fcgrt:ENSMUST00000210642 | down |
| mmu-miR-210-3p | up | Slc38a6:ENSMUST00000153941 | down |
| mmu-miR-376b-3p | up | Sult2a8:ENSMUST00000209425 | down |
| mmu-miR-423-5p | up | Cyp2u1:ENSMUST00000106337 | down |
| mmu-miR-485-5p_R+1 | up | Pgrmc2:ENSMUST00000058578 | down |
| mmu-miR-540-3p_R+2 | up | Ttc39c:ENSMUST00000025294 | down |
| mmu-miR-874-3p_R+1 | up | Acox1:ENSMUST00000066587 | down |
| mmu-miR-186-5p | up | Acox1:ENSMUST00000072948 | down |
| mmu-miR-200b-3p | up | Apon:ENSMUST00000060782 | down |
| mmu-miR-200c-3p | up | Akt2:ENSMUST00000051356 | down |
| mmu-miR-223-3p_R+1 | up | Prdx2:ENSMUST00000164807 | down |
| mmu-miR-25-3p | up | Slc22a28:ENSMUST00000065651 | down |
| mmu-miR-294-3p | up | Mup20:ENSMUST00000074018 | down |
| mmu-miR-29a-5p_R+1 | up | Mup3:ENSMUST00000107472 | down |
| mmu-miR-425-5p_R-1 | up | Mup3:ENSMUST00000107488 | down |
| mmu-miR-429-3p | up | Acat2:ENSMUST00000159697 | down |
| mmu-miR-467a-5p | up | Ptp4a2:ENSMUST00000165853 | down |
| mmu-miR-22-3p | up | Clcn2:ENSMUST00000007207 | down |
| mmu-miR-376b-5p | up | Klhdc3:ENSMUST00000071841 | down |
| mmu-miR-3535_L-1 | up | Mup1:ENSMUST00000135953 | down |
| mmu-miR-494-3p_R+1 | up | Elovl3:ENSMUST00000043739 | down |
| mmu-miR-326-3p | up | Cyp4a12a:ENSMUST00000084343 | down |
| mmu-miR-330-5p_R-1 | up | Cyp4a12b:ENSMUST00000094887 | down |
| mmu-miR-3473b_R-2 | up | Hist1h2bg:ENSMUST00000079251 | down |
| mmu-miR-3473e_R-3 | up |  |  |
| mmu-miR-125a-3p_R-1 | up |  |  |
| mmu-miR-1291_R-1 | up |  |  |
| mmu-miR-221-3p_R-1 | up |  |  |
| mmu-miR-222-3p_R+2 | up |  |  |

**Table S5. Results of homology comparison between mouse and human**

| query | subject | % identity | gene_id | gene_name | Description | trans_type | e-value | bit score |
| --- | --- | --- | --- | --- | --- | --- | --- | --- |
| 1700023H06Rik-ENSMUST00000161006 | chr5 | 89.58 | ENSG00000281357 | ARRDC3-AS1 | ARRDC3 antisense RNA 1 [Source: HGNC Symbol;  Acc: HGNC: 44145] | lncRNA | 1E-119 | 436 |
| Rian-ENSMUST00000182981 | chr14 | 100 | ENSG00000251363 | LINC02315 | long intergenic non-protein coding RNA 2315 [Source: HGNC Symbol; Acc: HGNC: 53234] | lncRNA | 0.0000002 | 63.9 |
| Rian-ENSMUST00000182981 | chr14 | 100 | ENSG00000258969 | LINC02307 | long intergenic non-protein coding RNA 2307 [Source: HGNC Symbol; Acc: HGNC: 53226] | lncRNA | 0.0002 | 54 |
